# Bedside assessment of swallowing in elderly subjects using psychotropic drugs

**DOI:** 10.1590/S1808-86942011000400019

**Published:** 2015-10-19

**Authors:** Marisa Portes Fioravanti, Fernanda Balero Miyahara, Heloisa Helena Cavallari, Onivaldo Bretan

**Affiliations:** 1 Master's degree, speech therapist; 2 Undergraduate student at the Botucatu Medical School (Faculdade de Medicina de Botucatu - UNESP); 3 Undergraduate student at the Botucatu Medical School (Faculdade de Medicina de Botucatu - UNESP); 4 Assistant professor; associate professor

**Keywords:** aged, psychotropic drugs, deglutition disorders

## Abstract

**Abstract:**

Neuroleptic drugs are used in several mental disorders, but are suspected of causing oropharyngeal dysphagia, mainly in the elderly.

**Aim:**

To study the effect of neuroleptic agents on swallowing of institutionalized older people.

**Material and method:**

A cross sectional study of swallowing in 47 subjects that either used or did not use neuroleptic drugs. Bedside swallowing tests with foods of four different consistencies were carried out.

**Results:**

There was no significant difference between the two groups. Users of neuroleptic medications showed a higher percentage of multiple swallowing while non-users had a higher percentage of oral food escape.

**Conclusion:**

Neuroleptic agents alone do not affect the mechanism of swallowing in the elderly; nonetheless. Further studies with a larger number of individuals and specific swallowing tests are needed.

## INTRODUCTION

The elderly often have systemic and neurologic conditions, and a variety of mental disorders that require medication^1,^^2^. Anxiolytic, antidepressant, antiepileptic, and antipsychotic drugs may affect cognition and alertness or act upon the brainstem, thereby causing or facilitating oral and/or pharyngeal phase swallowing disorders^3^, ^4^, ^5^. Neuroleptic antipsychotic drugs in particular have been implicated as causing asphyxia and silent dysphagia in psychiatric patients, particularly hospitalized elderly patients diagnosed with schizophrenia, bipolar or schizoaffective disorders, and depression with psychotic symptoms^6^, ^7^, ^8^, ^9^, ^10^. The term asphyxia has been used to describe respiratory disorders during meals in patients given neuroleptic antipsychotic drugs^5^,^8^, ^9^, ^10^. Although the word asphyxia denotes respiratory pathway obstruction, it does not clarify its mechanism, which may be other than a false route taken by the food bolus during swallowing. These reports are clinical observations only that do not explore the underlying mechanism of obstruction.

Studies applying specific swallowing tests on patients using psychiatric drugs are sparse. Videofluoroscopy of swallowing in psychiatric patients with choking found dysphagia in 18 of 21 subjects with pyramidal symptoms secondary to neuroleptic use^11^. A functional clinical test of swallowing for screening purposes was carried out in a similar study of diseases and drugs but in which patients had no history of choking; this study found alterations in 30% of subjects^7^. The consistency of water only was used in these tests, and subjects were of several age groups. There are no studies in the literature about the effect of psychotropic drugs on swallowing in elderly patients. Errors by caretakers during meals and the use of drugs to control behavioral changes are known to contribute to dysphagia, anorexia, pneumonia, and weight loss in persons living in nursing home residences^1,^^2^,^11^, ^12^, ^13^, ^14^, ^15^, ^16^. The purpose of this study was to observe the effect of neuroleptic drugs on the oropharyngeal swallowing phase in institutionalized elderly patients by carrying out functional clinical tests of swallowing with foods of different consistencies.

## SERIES AND METHODS

The institutional review board approved this study (387/2003). Participants were informed and signed a free informed consent form. The sample comprised 47 elderly patients living in a nursing home residence. The ages ranged from 61 to 91 years (mean - 78.5 years); there were 30 female and 17 male subjects. Of these 47 subjects, 29 (68%) were aged 75 years or above. Thirteen patients had systemic organic diseases and were medicated accordingly, 14 had systemic or neurologic conditions (Parkinson's disease, epilepsy) and were medicated accordingly and with psychiatric drugs, 12 were taking only psychiatric medication, and 8 subjects presented no diseases and did not take regular medication. Most patients with systemic diseases presented more than one disorder - mostly arterial high blood pressure and diabetes. Twenty-six patients took one or more psychiatric drugs; of these, 18 were given neuroleptics for several psychoses. The 26 subjects were also given anxiolytic, antidepressant, antiepileptic, and antiparkinsonian drugs. The exclusion criteria were as follows: subjects that were unable to carry out the examinations because of physical or mental handicaps, and subjects with uncontrolled acute or chronic infectious diseases. Table 1 shows the neuroleptic drugs that were given to patients.

**Table 1:** Prescribed neuroleptic drugs.

Generic name of the drug	Number of subjects (n=18)
Haloperidol	6
Chlorpromazine	5
Pericyazine	4
Thioridazine	1
Levomepromazine	1
Risperidone	1

## METHODS

The functional clinical examination of swallowing were based on the protocols proposed by Logemamn^17,^^18^ and Perlman & Schulze-Debrieu^19^.

Four food consistencies were offered: solid (biscuit), thick and thin pasty (diet juice and food thickeners), and liquid (water-diluted juice). The volume of pasty and liquid foods was 5ml. The following signs were annotated: altered chewing, absent or uncoordinated masticatory movements; anterior leakage, or inability to avoid drooling; multiple swallowing, more than two perceived swallows; altered elevation of the larynx, assessed by the evaluator placing the index and middle fingers between the larynx and the hyoid bone and noting if it elevated; presence of coughing before, during, or just after swallowing; and altered voice quality after swallowing, noted when voice was perceived as “wet”^20^. The signs altered elevation of the larynx, coughing, and altered voice were considered as highly indicative of dysphagia and risk of aspiration^20^. The elderly subjects were allocated to two groups: group 1 comprised 18 subjects that were taking neuroleptic drugs; group 2 comprised 29 subjects that did not take neuroleptic drugs. There were 47 elderly subjects in total. There were elderly patients taking antidepressant, anxiolytic, antiparkinsonian, and antiepileptic drugs in both groups. The statistical analysis sought possible factors or confounding variables and/or interactions on the outcomes using odds ratio estimates and the chi-square test or Fisher's exact test to estimate the effects of gender, age, arterial hypertension, and diabetes on the effects that were investigated. Fisher's exact test was then applied to compare the groups. Differences and effects were considered statistically significant when *p* < 0.05.

## RESULTS

The statistics did not reveal interaction or confounding factors or variables that might affect the results and compromise the veracity of possible findings. There were no differences between the groups. A non-statistically significant percentage difference between groups was seen in anterior leakage and multiple swallowing (Table 2). Multiple swallowing was found at a higher percentage in non-users of neuroleptic drugs, while anterior leakage was more frequent in the other group. Table 3 shows how each clinical sign was distributed among subjects in both groups.

**Table 2:** Percentage of signs that were encountered.

	Groups		
Clinical sign	1	2	*p*
Altered voice	16.7	17.2	1.000(1)
Coughing	11.1	6.9	0.631(1)
MC	5.6	3.4	1.000(1)
Altered chewing	72.2	65.5	0.632(2)
Anterior leakage	22.2	13.8	0.691(1)
Multiple swallowing	16.7	27.6	0.492(1)
Altered elevation of the larynx	16.7	13.8	1.000(1)

(1) chi-square test

(2) Fisher's exact test

**Table 3:** Clinical signs in both groups.

	Patient no.	Altered chewing	anterior leakage	MC	multiple swallowing	altered elevation of the larynx	coughing	altered voice
Taking neuroleptics	1							
	2							
	3							
	4							
	5							
	6							
	7							
	8							
	9							
	10							
	11							
	12							
	13							
	14							
	15							
	16							
	17							
	18							
Not taking neuroleptics	19							
	20							
	21							
	22							
	23							
	24							
	25							
	26							
	27							
	28							
	29							
	30							
	31							
	32							
	33							
	34							
	35							
	36							
	37							
	38							
	39							
	40							
	41							
	42							
	43							
	44							
	45							
	46							
	47							

## DISCUSSION

Studies on the relationships among neuroleptics, nutrition, dysphagia, and asphyxia have all been clinica^5,^^6^,^8^, ^9^, ^10^. Two studies applied specific swallowing evaluation methods in subjects taking neuroleptics and found disorders in a significant number of their sample^7,^^11^. These studies, however, were not controlled and patients belonged to several age groups, including elderly subject; the latter have specific physical features and systemic diseases that may lead to dysphagia^8^, ^9^, ^10^. Furthermore, the authors did not describe the clinical signs in those patients, and used only a watery consistency without informing the volume^7,^^11^. Dysphagia and aspiration may occur depending on the volume, the amount of food, and its consistency in the mouth^19^, ^20^, ^21^, ^22^, ^23^. Thus, differences in the results of the present study and other published papers are due both to the methods for evaluating swallowing and to observing carefully the relationship between food consistency and clinical signs^5^, ^6^, ^7^, ^8^, ^9^, ^10^, ^11^,^19,^^21^,^23^, ^24^, ^25^. Therefore, while one of those studies found changes in 30% of subjects, and another found changes in 90% of subjects, we encountered no significant effect of neuroleptic drugs on swallowing^7,^^11^. As other factors and co-morbidities may cause dysphagia, even in youths, previous reports may have attributed swallowing effects to neuroleptic drugs without first applying appropriate verification methods. On the other hand, those papers suggested an association between drugs and dysphagia which cannot be neglected. Thus, it is wise to observe and refer patients to specialists in swallowing, since other risk factors may also be present^26,^^27^. It is worth noting that, aside from evident signs of dysphagia such as choking, respiratory difficulties, and coughing during swallowing, observing these patients may uncover weight loss of no apparent cause and/or recurring pneumonia by silent aspiration^28^.

Although no difference was found between the groups, this study showed that the percentages of OM and anterior leakage were higher, albeit in different groups. These signs may be encountered in elderly patients without swallowing difficulties or in subjects with difficulty in the oral phase^21^. Anterior leakage may be associated with some other alteration in the mouth that could increase the inefficiency of the oral phase^23^. Altered chewing is also frequent in edentulous patients or with dental appliances; in such cases, poorly performing oral muscles also alter chewing.^26,^^27^ A previous study of the same group of elderly patients showed that about 40% had one or more of the three clinical signs suggesting passage of the food bolus into airways, coughing, altered elevation of the larynx, and altered voice with one or more food consistencies^20^. In the present study, the percentage was not higher in the group of patients that were given neuroleptic drugs, suggesting that these drugs were not in themselves causative of the signs that were found.

This study is limited by the small sample; the findings do not allow us to generalize. Drug dosages and duration of use were also not presented, so we can only speculate about the effects on swallowing of drug dosages and duration of use.

## CONCLUSION

Neuroleptic drugs by themselves do not affect swallowing in institutionalized elderly subjects. Further studies with larger samples are needed to perform detailed clinical studies of swallowing; these studies need to take into account the medication dose and duration of use, and should present these findings thoroughly. Any effect that these drugs may eventually have on the oropharyngeal phase of swallowing may be more accurately seen.
